# A Validated RP-HPLC Method for Monitoring Pollutants Removal during Microalgae Bioremediation of Polluted Waters

**DOI:** 10.1007/s00128-025-04085-y

**Published:** 2025-07-29

**Authors:** Bruna Santos, Juliana Araújo, Filomena Freitas, Abílio J. F. N. Sobral, Telma Encarnação

**Affiliations:** 1 i4HB – Institute for Health and Bioeconomy, School of Science and Technology, NOVA University Lisbon, 2829-516 Caparica, Portugal; 2 UCIBIO – Applied Molecular Biosciences Unit, Department of Chemistry, School of Science and Technology, NOVA University Lisbon, 2829-516 Caparica, Portugal; 3 PTScience, Alcobaça, Portugal; 4 CQC-IMS, Coimbra Chemistry Center - Institute of Molecular Sciences, University of Coimbra, 3004-535 Coimbra, Portugal

**Keywords:** RP-HPLC, Method development, Validation, Contaminants of emerging concern, Microalgae bioremediation

## Abstract

A Reversed-Phase High-Performance Liquid Chromatography (RP-HPLC) method was developed and validated to simultaneously quantify six contaminants of emerging concern (CECs) in the context of microalgae bioremediation. RP-HPLC was chosen for its ability to separate substances of differing polarities, its use of cost-effective and low-toxicity mobile phases, and the capacity to selectively adjust pH for enhanced sample resolution. To validate this method, standard guidelines were followed to evaluate system suitability, linearity, accuracy, precision, recovery, limits of detection and quantification, selectivity and specificity. Limits of detection and limits of quantification were, respectively, 0.017 and 0.051 µg mL^−1^ for paracetamol (PAR), 0.024 and 0.072 µg mL^−1^ for methylparaben (MP), 0.008 and 0.027 µg mL^−1^ for imidacloprid (IMID), 0.014 and 0.041 µg mL^−1^ for bisphenol A (BPA), 0.023 and 0.069 µg mL^−1^ for triclosan (TCS) and 0.016 and 0.048 µg mL^−1^ for ibuprofen (IBU). Precision and accuracy values assessed inter-day and intra-day were all within the acceptance limits of relative standard deviation (%RSD) and %bias of less than 15%. Percentage recovery was determined to be within the acceptable range of 80–120%, except for TCS due to low solubility. Applicability was demonstrated by assessing the effect of contaminant exposure to microalgae cells, and contaminant removal. The developed method is linear, precise and accurate and has been successfully applied to the quantification of the six contaminants during microalgae bioremediation.

**Electronic supplementary material:**

The online version of this article (10.1007/s00128-025-04085-y) contains supplementary material, which is available to authorized users.

## Introduction

Contaminants of Emerging Concern (CECs) are natural or synthetic substances for which there is limited information available or no current standard regulations regarding their presence in the environment (Morin-Crini et al. 2022). Some CECs can be considered as ubiquitous, since they are found in air, soil, water, food, food chain and humans (chemical burden) (Tarafdar et al. 2022; Wang et al. 2024; Yadav et al. 2021). They pose substantial dangers to ecosystems and human health since they have unfavourable characteristics, including high polarity, bioaccumulation, persistence in the environment and resistance to biodegradation (Maryjoseph and Ketheesan 2020).

CECs can be divided into different categories of chemicals, including persistent organic pollutants (POPs), pharmaceuticals and personal care products (PPCPs), pesticides, microplastics, and other industrial products. Many of these compounds are carcinogens, mutagens or can act as endocrine disruptors (EDs) (Morin-Crini et al. 2022). Are of particular concern since their structure mimics natural hormones.

EDs interfere with different signalling pathways by interacting with receptors in the organism, deregulating the endocrine system. They affect humans and wildlife, causing mainly reproductive disruption, increased risk of cancer (breast, prostate, ovarian, thyroid), delay in neurological development, etc. (Modica et al. 2022).

In this study, six CECs were selected as representatives of different categories of CECs. Additional characteristics were considered including their toxicity, occurrence and persistence in the environment, and/or endocrine disrupting activity. They are bisphenol A (BPA), imidacloprid (IMID), methylparaben (MP), triclosan (TCS), paracetamol (PAR) and ibuprofen (IBU). These chemicals originate from different sources since they are used for diverse applications, nevertheless, when released in the environment, they all share the potential to cause harm to humans and wildlife.

BPA is a monomer widely used to produce polycarbonate plastics, being found in windows, eyewear and epoxy resins used for coating (Tarafdar et al. 2022). It is identified at European Union (EU) level as an endocrine disruptor as it mimics estrogenic activity and represents a great concern since it is one of the most commonly detected micropollutants in the environment (Ferrer-Polonio et al. 2021; Li et al. 2009). On the other hand, IMID is a systemic insecticide part of the chemical class of neonicotinoids, which are commonly used in agriculture to control diverse pests, and indoor for animal parasite control in pets such as dogs and cats. Its excessive use leads to accumulation and increases its occurrence in the environment, where it is acutely toxic and deadly to beneficial species, including honeybees and aquatic organisms (Bhende and Dafale 2023; Merga and Van Den Brink 2021). MP is a widely used preservative intended to prevent mould and bacteria in food, cosmetics, and pharmaceuticals (Bolujoko et al. 2022). Its presence in surface waters is widely reported, and concerns about potential toxic effects on aquatic organisms are being raised (Puerta et al. 2020). TCS, in turn, is a broad-spectrum antimicrobial chemical used in self-care products such as soaps, hand sanitizers, and toothpaste (Dhillon et al. 2015). Harmful effects of TCS are reported in microorganisms and aquatic life due to bioaccumulation, biomagnification and ED activity (Gupta et al. 2024). These two last contaminants are identified as potential EDs at EU level. PAR and IBU are two nonsteroidal anti-inflammatory drugs (NSAIDs) that do not require medical prescription, thus having high human consumption rates. Consequences of their excessive consumption include accumulation in water bodies and negative effects on aquatic life due to long exposure to low concentrations (ng-µg L^−1^) (Żur et al. 2018). For instance, Guiloski et al. (2017) reported that PAR at 0.25–2.5 µg L^−1^ causes endocrine disruption and hepatotoxicity in the fish *Rhamdia quelen*. In the case of IBU, its exposure to zebra fish for 7 days resulted in lipid peroxidation and disruption of enzymatic activity (Jan-Roblero and Cruz-Maya 2023). The structures of the six CECs and some of their characteristics are presented in Table 1.

**Table 1:** Chemical structures and characteristics of the selected CECs relevant to this method’s validation. ªValues were accessed in PubChem database

CEC	Structure	Category of CEC	Solubilityª (mg L^− 1^)	Effluent concentration (µg L^− 1^)	References
MP		Preservative	2500	0.011	Khan et al. (2022)
TCS		Antimicrobial	10	0.734	Khan et al. (2022)
BPA		Industrial chemical	120	0.123	Khan et al. (2022)
IMID		Pesticide	610	0.300	Merga and Van Den Brink, (2021)
PAR		Pharmaceutical	14,000	0.020–15	Al-Kaf et al. (2017)
IBU		Pharmaceutical	21	0.100–11	Jan-Roblero and Cruz-Maya (2023)

Current wastewater treatment techniques are unsuitable for effectively removing CECs from the environment, which are increasingly being detected in surface water. The use of microalgae in wastewater treatment is not new and has been exploited for decades. However, the main focus has been on nutrient removal (nitrates and phosphates) (Encarnação et al. 2023). Fewer studies have focused on microalgae remediation of CECs, mainly because only more recently CECs have been recognized as a major environmental issue (Sauvé and Desrosiers 2014). The ability of microalgae to remove various CECs has now been successfully studied; thus, further exploring its potential is of great relevance (Maryjoseph and Ketheesan 2020).

To ensure robust and reliable quantification of the selected CECs in the bioremediation studies, a chromatographic technique (RP-HPLC) and validated methods were employed. Some of the advantages of RP-HPLC technique include the ability to analyse a broad range of compounds with different polarities and molecular masses, the use of low toxicity solvents and small sample sizes with accurate results (Ankush Bhalerao et al. 2023). This technique and these methods are similar to those rigorously employed by many industry sectors, as these methods adhere to established quality standards and follow certified guidelines.

*Nannochloropsis* sp. was the microalgae selected for this study because of its rapid growth rate, resilience under unfavourable conditions, and interesting biomass composition, particularly lipid productivity (Mohy El-Din 2020). The CECs concentrations were chosen to assess the resilience and effectiveness of *Nannochloropsis* sp. remediation of in a pollution scenario. While the CECs values often found in the environment are in the order of magnitude of ng L^−1^ to µg L^−1^ (Khan et al. 2022), there have been reports of specific CEC at higher concentrations ranging from less than 1 ng mL^−1^ up to 17 µg mL^−1^ (Mompó-Curell et al. 2024). Such high values can occur due to industrial wastewater discharges, accidental spills, or even environmental accumulation. Therefore, to assess the maximum bioremediation potential and test the limits of bioremediation under controlled conditions, high values of concentrations of pollutants were used. While the bioremediation study, which involved the development of a validated method, provides knowledge with respect to resilience and quantifies the amount of pollutants removed in such a scenario, it also provides baseline data for optimising conditions and parameters before transitioning to more realistic scenarios or upscaling bioremediation systems.

## Material and Methods

### Reagents and Chemicals

The CECs relevant to this study were obtained from different suppliers. PAR and TCS were purchased from Fagron Iberica (Spain) and IBU was acquired from Laboratórios Medinfar (Lisboa, Portugal). IMID was supplied by EHRENSTORFER™ and BPA was supplied by Sigma-Aldrich. MP was purchased from Tokyo Chemical Industry. Varicon Aqua Solution (Malvern, UK) provided the microalgae *Nannochloropsis* sp. and the culture medium f2 Cell-hi TEViT. The remaining reagents and solvents were of analytical or HPLC grade.

### Instrumentation

The HPLC analysis of the six CECs was conducted using a Dionex Ultimate 3000 system supplied with an auto-injector and a diode array detector (DAD) with four variable UV/Vis dual-wavelength detectors. The column selected for the analysis was a Kinetex® Biphenyl, Phenomenex® (Torrance, USA), with 5 µm particle size, 100 Å pore size, 4.6 mm internal diameter, and 250 mm length, supported with a SecurityGuard™ cartridge Phenomenex® (Torrance, USA), with 4.6 mm internal diameter, which was in an oven at a temperature of 40 °C. The data were recorded and processed using Chromeleon™ 7 software on Windows™ 10. Chromatographic analysis was conducted in multistep gradient mode, as indicated in Table 2.

**Table 2:** Chromatographic conditions of the gradient HPLC method

Time (min)	Eluent A (%)	Eluent B (%)	Flow rate (ml min^− 1^)
0	50	50	0.6
5	80	20	0.6
15	100	0	0.6
17	100	0	0.6

Preferentially, the UV detector was set at 275 nm for the simultaneous detection of IMI, BPA, TCS, PAR, and MP, and a wavelength of 220 nm was selected to quantify IBU. The injection volume was 20 µL for standard and samples. Before analysis, every standard and sample were filtered through 0.22 µm syringe filters. In order to guarantee a rapid method, without affecting separation quality and preserving the peak resolution, the best equilibration time was found at 2 min, with 50% of eluent B was performed before every injection. A run time of 17 min was found adequate for the separation of the six analytes, with a 2 min washing step with buffer between runs.

### Preparation of Mobile Phase, Stock and Standard Solutions and Quality Controls

The mobile phase comprised two eluents: an organic solvent and a buffer solution. The organic solvent used was methanol (CHROMASOLV®, gradient grade, for HPLC, ≥ 99.9%) from Sigma-Aldrich® (34,885) with 5 mM of ammonium acetate (≥ 99%), from Riedel-de Haën (32,301), with 0.1% of acetic acid (≥ 99.7%), from PanReac AppliChem ITW Reagents (131,008.1211), prepared by adding 154 mg of ammonium acetate and 380 µL of acetic acid to 400 mL of methanol. The pH of eluent A was 6.06 ± 0.01. Eluent B was prepared by adding 154 g of ammonium acetate and 380 µL of acetic acid to 400 mL of ultrapure water, with a pH of 3.87 ± 0.01. The mobile phase was filtered through a 0.22 µm nylon membrane filter acquired from FILTER-LAB® (MNY022047N) and sonicated to degas.

Six stock solutions of 1 mg mL^−1^ of the CECs were prepared by dissolving each one of them in the mobile phase (MeOH:buffer 1:1). A similar solution containing the mixture of the six analytes at the same concentration was prepared. The various concentrations used throughout the validation method were obtained by dilution of the stock solutions (1 mg mL^−1^). To determine limits of detection and quantification, six standard solutions were prepared ranging from 0.1 to 1.25 µg mL^−1^. Similarly, to obtain a calibration curve, eight standard solutions were prepared with concentrations ranging from 0.5 to 100 µg mL^−1^. Quality controls of 1, 50 and 100 µg mL^−1^ were prepared to evaluate accuracy and precision intra and inter-day. All samples were filtered through a PTFE 0.22 µm syringe filter from OlimPeak®, Teknokroma® (TR-200503) before injected in the HPLC system.

### Method Validation

Method validation is the process of confirming the consistent capabilities of the method for the required application. The developed method for quantification of IMID, BPA, TCS, MP, PAR and IBU followed the US Food and Drug Administration (FDA), Eurachem, and the International Conference on Harmonization (ICH) guidelines for method validation. The estimated characteristics were system suitability (SS), limits of detection (LOD) and quantification (LOQ), linearity, accuracy, precision, recovery, and selectivity and specificity. The equations used to calculate each SS parameter are given in table S1. Equations 1 and 2 were used to calculate LOD and LOQ, respectively, where σ is the standard deviation of the response, and S is the slope of the calibration curve (ICH 2022).

1 $$LOD=\frac{3.3\times \sigma }{S}$$

2 $$LOQ=\frac{10\times \sigma }{S}$$

### Method Applicability

#### *Nannochloropsis* sp. Culture Conditions

The microalgae *Nannochloropsis* sp. was purchased from Varicon Aqua Solution, Malvern, UK, and was grown for 11 days in 2 L flasks with f2 culture medium. A volume of 200 mL of the *Nannochloropsis* sp. culture, with approximately 2.2 × 10^6^ cell mL^−1^ were filtered and washed with distilled water. Cells were subsequently transferred to a sterilized 250 mL Erlenmeyer flask, to which 200 mL of f2 culture was added (Varicon Aqua Solution, Malvern, UK) were added.

#### Removal of CEC from Water by *Nannochloropsis* sp.

**Fig. 1:**
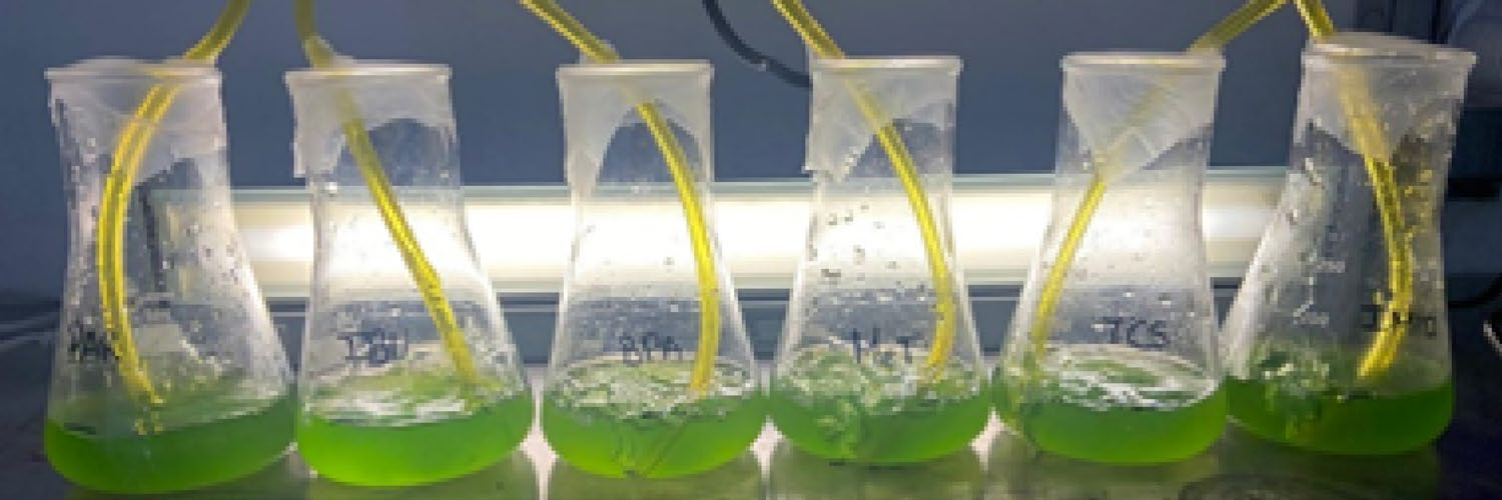
Individual experiments using each CEC at 50 µg mL^−1^

A concentration of 10 μg mL^−1^ of IMI, BPA, MP, IBU and PAR, and 0.5 μg mL^−1^ of TCS (poorly soluble in water), were added to the *Nannochloropsis* sp. culture. Similarly, the same concentration of each pollutant, IMI, BPA, TCS, MP, IBU and PAR was added to 200 mL of culture medium f2, and to distilled water, both without cells. A negative control of *Nannochloropsis* sp. culture was also monitored. Each experiment was carried out in triplicate. The experiments were kept at room temperature under a light intensity of 50–60 µmol m^−2^ s^−1^ with 24:0 photoperiod for 8 days. The cultures were aerated by bubbling atmospheric air, at a rate of 1500 cm^3^ min^−1^. Distilled water was added when needed to ensure the same volume due to water loss by evaporation. Samples of 5 mL were collected every 24 h, and stored at − 19 °C until analysis. Similarly, preliminary studies were performed using 25 μg mL^−1^ and 50 μg mL^−1^ of each pollutant in the mixture (20 and 22.5 μg mL^−1^ of TCS, respectively), to understand the effect of extreme concentrations on growth. In addition, experiments with individual pollutants at 50 μg mL^−1^ were also tested (22.5 μg mL^−1^ for TCS) (Fig. 1). The results were then analyzed using the developed RP-HPLC method, after the microalgae cells were filtrated from the experimental solution using PTFE 0.22 µm syringe filters. The cell density was assessed by cell counting using a Neubauer chamber.

## Results and Discussion

### Method Development

RP-HPLC is a widely used analytical method used to separate, identify, and quantify multiple components within a mixture. Although it is a versatile technique, the development and validation of a method might be challenging, given the necessity to carefully control multiple variables to achieve a desired separation. These include the column, mobile phase, pH, flow rate, column temperature, wavelength, etc. Our recent study reported a RP-HPLC method for the simultaneous analysis of PAR, IBU, olanzapine, simvastatin and simvastatin acid, which was used as foundation for the current methodology (Encarnação et al. 2020).

A Biphenyl column was selected for the analysis of the six analytes. This column is characterized by high selectivity for compounds with aromatic rings, which interact by π–π bonds with the phenyl-based stationary phase. This motivated the choice of this column since the six analytes contain, at least, one aromatic ring. The composition and pH of the mobile phase were also considered. First, a mobile phase of acetonitrile:water was studied, however, the resulting peaks showed low resolution. Thus, methanol was chosen as organic solvent, working as eluent A, allowing for well-resolved and separated peaks. Ultra-pure water was selected as eluent B. To control pH, a buffer was tested considering the optimum pH range for peak separation. Therefore, the addition of 5 mM ammonium acetate with 0.1% acetic acid to the mobile phase was found to be experimentally effective for the separation of the six analytes. A flow rate of 0.6 mL min^−1^ was adequate for this method since methanol in the mobile phase slightly increases its viscosity.

The temperature of the oven column was tested at room temperature and at 40 °C. Reduced retention times were obtained at 40 °C, compared to those at room temperature, which is beneficial for the process throughput. In addition, sharper peaks with improved resolution were also observed at this temperature, which favoured the decision to select 40 °C as the optimal temperature. A wavelength of 275 nm was found to be optimal and specific for most of the analytes, except for IBU. Consequently, a wavelength was defined for the analysis of IBU alone, 220 nm, at which its peak exhibited the greatest absorptivity.

The development of a gradient method was preferred to separate the six analytes over an isocratic method, due to the complexity of the mixture, which contains components with varying polarities and affinities for the stationary phase. In the development of the gradient method, compounds with higher polarity, such as PAR, elute first. With the increasing of the organic content in the mobile phase, moderately nonpolar compounds like MP, IMID, BPA, and IBU elute sequentially. Components with non-polar structures are the last to elute, when the mobile phase is mostly methanol, as in the case of TCS. The optimal separation was achieved for the gradient method described in Table 2.

### Method Validation

#### System Suitability

**Fig. 2:**
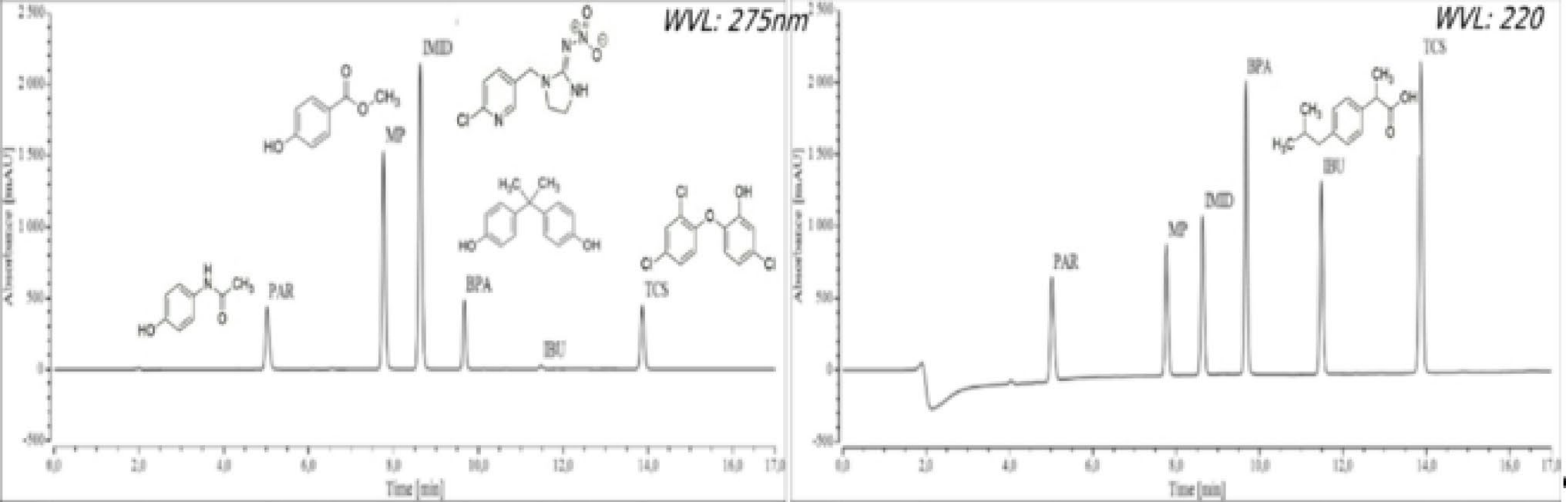
Chromatograms of the standard solution of the mixture at 75 µg mL^−1^, obtained to evaluate the system suitability of the developed method. A wavelength of 220 nm was used for IBU, and 275 nm was set to evaluate PAR, MP, IMID, BPA, and TCS

The SS test revealed that the chromatographic system is suitable for the identification of PAR, MP, IMID, BPA, TCS, and IBU (Fig. 2). The parameters evaluated in the SS test are all within the acceptable values defined by the guidelines (Tables S1 and S2). The %RSD is lower than 2 % for both retention time and peak area values, demonstrating the suitability of this system for the detection of the six analytes. The values were higher than 2 for resolution, indicating well-separated peaks for the six compounds. In addition, the tailing factor values lower than 2 are indicative of symmetric peaks. The column was also proven efficient, with a value of the number of theoretical plates superior to 1000.

The interpretation of a capacity factor value when working in gradient mode can be complex due to the continuous change in mobile phase composition, therefore, its calculation is different from that in isocratic mode (Snyder et al. 1983). The value obtained for PAR is below the acceptable limit that is generally considered for isocratic mode, which, in this case, has no theoretical significance.

#### LOD and LOQ

The estimated LOD and LOQ are shown in Table 3. These values are characteristic of a highly sensitive method, and adequate for detecting and quantifying the six analytes in the bioremediation studies. Such results obtained in this study are similar or in the range (µg mL^-1^-µg L^-1^) of those reported in the literature for other RP-HPLC methods (Aminu et al. 2018; Encarnação et al. 2021, 2020; Javed et al. 2023; Radu et al. 2016).

**Table 3:** Results obtained from the linear regression using the least squares method, the corrected calibration curves calculated using the weighted least squares linear regression model, and LOD and LOQ for the six analytes

Analyte	R^2^	Slope ± SE	Intercept ± SE	Corrected Calibration Curve	LOD (µg mL^− 1^)	LOQ (µg mL ^− 1^)
PAR	0.9999	0.594 ± 0.002	0.123 ± 0.080	y = 0.603*x* + 0.012	0.017	0.051
MP	0.9997	1.700 ± 0.010	0.654 ± 0.480	y = 1.748*x* + 0.049	0.024	0.072
IMID	0.9986	2.497 ± 0.035	1.706 ± 1.614	y = 2.607*x* + 0.073	0.009	0.027
BPA	0.9999	0.474 ± 0.001	0.045 ± 0.038	y = 0.478*x* + 0.004	0.014	0.041
TCS	0.9999	0.559 ± 0.001	0.034 ± 0.037	y = 0.560*x* + 0.007	0.023	0.069
IBU	0.9999	1.488 ± 0.007	0.315 ± 0.313	y = 1.514*x* – 0.048	0.016	0.048

#### Linearity

A calibration curve was obtained by linear regression using the least squares method. Values of correlation coefficient, slope, and intercept were accessed and summarized in Table 3. Values of R^2^ greater than 0.998 indicate that there is a good linear relationship between chromatogram areas and concentrations (Shabir et al. 2007) However, the need to cover wide concentration ranges, results in heteroscedasticity across the calibration range, which interferes with the prediction of lower concentrations. For that reason, the calibration curve was corrected using a weighted least squares linear regression model to improve the quality of the analytical results (Table 3).

#### Accuracy and Precision

The accuracy and precision of the method were evaluated as %bias and %RSD, respectively, intra-day and inter-day. According to the validation guidelines, this method is significantly accurate, reliable, and reproducible, as the results for the six analytes are all below 15% for %RSD and %bias (U.S. Food and Drug Administration 2018).

#### Selectivity and Specificity

The results show that the developed method is selective for the six analytes, at the two relevant wavelengths (Figure S1). The elution time of the f2 medium was obtained at 4 min, indicating that there is no matrix interference on the elution time of the six analytes.

#### Recovery

Recovery percentages were all within the acceptable range of 80–120%, which means that the method is adequate to simultaneously quantify PAR, MP, IMID, BPA, and IBU present in the f2 medium (Table S3) (ICH 2022). On the other hand, TCS recovery values were significantly lower than the acceptable range, which can be explained by its low solubility in water (10 μg mL^−1^). This results in great difficulty in solubilization, and potential aggregation. Moreover, other physicochemical characteristics of TCS, including hydrophobicity (logKow= 4.76) may also contribute to such results, making it prone to bind to surfaces (Kim et al. 2023). Some abiotic degradation of TCS is also possible, especially due to the presence of light and components of the f2 growth medium (EDTA, metal ions) (Singh and Kaur 2023).

#### Method Applicability

**Fig. 3:**
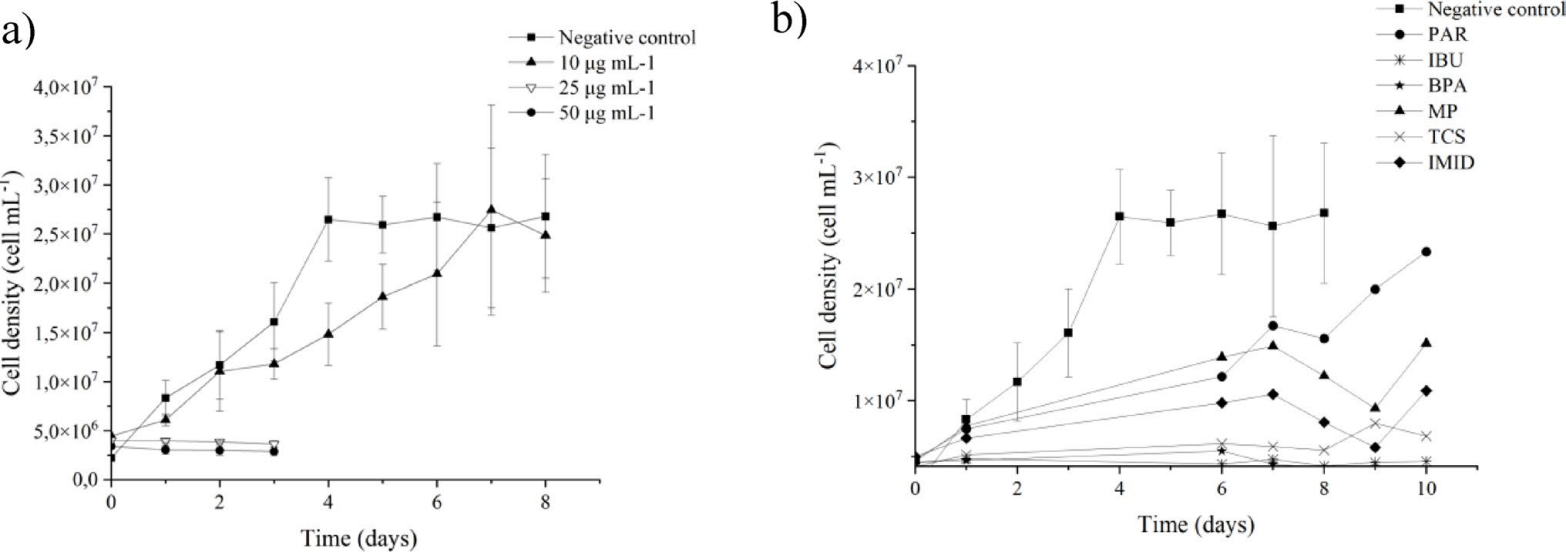
**a** Cell density of Nannochloropsis sp. in bioremediation experiments with 10, 25 and 50 µg mL^−1^ of each contaminant; **b** Cell density of Nannochloropsis sp. in individual experiments with each CEC at 50 µg mL^−1^. (Santos et al. 2025)

From preliminary studies, it was observed that the extreme CECs concentrations (25 and 50 µg mL^-1^) were toxic to *Nannochloropsis* sp., inhibiting cell growth and limiting bioremediation ability (Fig. 3a). Another preliminary study was performed using individual contaminants to understand if a particular polluant was responsible for causing major toxicity to the cells at 50 µg mL^−1^ (Fig. 3b). It was concluded that all CEC have a negative impact on cell growth at those concentrations, and that IBU, TCS and IMID were the most toxic. Therefore, a concentration of 10 µg mL^−1^ for the mixture was found adequate to perform the bioremediation studies.

*Nannochloropsis* sp. exposed to 10 µg mL^−1^ of each pollutant was able to achieve the same cell density as the negative control, despite having a slower growth rate. Results from HPLC analysis showed that a total removal of 2.36, 7.15, 1.50, 6.29 and 4.61 µg mL^−1^ of PAR, MP, IMID, BPA and IBU, respectively, was achieved in the experiments containing microalgae. The concentration of TCS during the experiment was found to be below the limits of detection and quantification, thus, results were inconclusive. This could be explained by its low solubility in water.

## Conclusion

A RP-HPLC method was developed and validated for the determination and quantification of PAR, MP, IMID, BPA, IBU, and TCS during bioremediation using microalgae. The method was shown to be sensitive, linear, precise, accurate, and selective. While this method is not directed to ambient environmental monitoring of the selected contaminants, where concentrations are typically much lower and matrixes complexity is significantly higher, it was developed and validated for studying microalgae bioremediation in spiked matrices at high concentrations.

Using HPLC-RP for microalgae bioremediation studies presents some disadvantages including the fact that dealing with biological and complex matrices can interfere with the analysis. The presence of microalgae endogenous compounds and the CECs biodegradation products derived from the bioremediation process can lead to inaccurate quantification, supressing or enhancing the signal, requiring additional critical analysis.

Nevertheless, HPLC-RP is a powerful technique for microalgae bioremediation studies, offering advantages such as versatility, precision, accuracy, and the ability to separate and quantify a wide range of CECs from moderately polar to non-polar. Another crucial advantage is its high robustness and reproducibility. An HPLC-RP method that has been developed and validated ensures consistent, reliable and reproducible results over time and across laboratories.

These advantages make a HPLC-RP a powerful analytical tool for understanding the extent of microalgae removal and degradation pathways and for evaluating microalgae treatment approaches.

This study also demonstrates that the developed HPLC-RP method is suitable to evaluate the bioremediation of CECs, and shows the potential of the species *Nannochloropsis* sp. for the removal of PAR, MP, IMID, BPA and IBU.

## Electronic supplementary material

Below is the link to the electronic supplementary material.

Supplementary file 1 (DOCX 19 kb)
