# Supplementary material for: A Validated RP-HPLC Method for Monitoring Pollutants Removal during Microalgae Bioremediation of Polluted Waters
Source: Bull Environ Contam Toxicol. 2025 Jul 29;115(2):22. doi: 10.1007/s00128-025-04085-y (PMC12307476; doi:10.1007/s00128-025-04085-y)
Supplement: Supplementary file 1 — (DOCX 19 kb) [file 128_2025_4085_MOESM1_ESM.docx]

**Supplementary material**

**Table S1:** Results of the SS test parameters.

|  | PAR |  | MP |  | IMID |  | BPA |  | TCS |  | IBU |  |
| --- | --- | --- | --- | --- | --- | --- | --- | --- | --- | --- | --- | --- |
|  | Peak Area (mAU min) | Retention Time (min) | Peak Area (mAU min) | Retention Time (min) | Peak Area (mAU min) | Retention Time (min) | Peak Area (mAU min) | Retention Time (min)) | Peak Area (mAU min) | Retention Time (min) | Peak Area (mAU min) | Retention Time (min) |
| Mean (n=6) | 44.53 | 5.01 | 128.61 | 7.75 | 191.81 | 8.62 | 35.61 | 9.66 | 41.89 | 13.86 | 113.32 | 11.47 |
| SD | 0.29 | 0.01 | 0.43 | 0.02 | 0.89 | 0.02 | 0.18 | 0.01 | 0.30 | 0.01 | 1.25 | 0.01 |
| %RSD | 0.65 | 0.25 | 0.33 | 0.21 | 0.47 | 0.18 | 0.51 | 0.10 | 0.71 | 0.05 | 1.10 | 0.07 |
| Plates (N) | 15348 |  | 54973 |  | 59266 |  | 110824 |  | 13942 |  | 12060 |  |
| Tailing factor (T) | 1.27 |  | 1.13 |  | 1.10 |  | 1.06 |  | 1.10 |  | 1.06 |  |
| Capacity factor (k') | 1.50 |  | 2.88 |  | 3.31 |  | 3.83 |  | 5.93 |  | 5.07 |  |
| Resolution (Rs) | 18.74 |  | 6.31 |  | 8.15 |  | 14.65 |  | 17.06 |  | 16.68 |  |

**Table S2:** Acceptance criteria for SS parameters.

| Parameter | Equation | Acceptance criteria | Reference |
| --- | --- | --- | --- |
| %RSD | $\%RSD=\frac{SD}{mean}\times100$ | ≤ 2.0% | (Épshtein, 2004) |
| Theoretical plates (N) | $N=5.54\times{(\frac{t_{R}}{W_{50\%}})}^{2}$ | > 1000 | (Épshtein, 2004) |
| Tailing factor (T) | $T=\frac{{RW}_{5\%}+{LW}_{5\%}}{2\times{LW}_{5\%}}$ | ≤ 2.0 | (A.M.T. Committee, 1994.) |
| Capacity factor (k') | $k'=\frac{t_{R}-t_{0}}{t_{0}}$ | > 2.0 | (A.M.T. Committee, 1994.) |
| Resolution (Rs) | $Rs=1.18\times\left\vert\frac{t_{RefPeak}-t_{R}}{W_{50\%RefPeak}+W_{50\%R}} \right\vert$ | > 2.0 | (A.M.T. Committee, 1994.) |

**Table S3:** Estimated values to evaluate the accuracy and precision of the method intra-day.

|  | Theoretical concentration (µg/mL) | | Intra-day (n=6) |  | |  | | | |
| --- | --- | --- | --- | --- | --- | --- | --- | --- | --- |
| Analytes |  |  | Measured concentration mean ± SD (µg/mL) | Precision %RSD | | Accuracy %BIAS | |  |  |
|  | 0.5 | 0.492 ± 0.005 | | | 1.01 | | -1.66 | |  |
|  | 1.5 | 1.510 ± 0.008 | | | 0.53 | | 0.65 | |  |
| **PAR** | 50 | 49.395 ± 0.626 | | | 1.27 | | -1.21 | |  |
|  | 100 | 97.805 ± 1.254 | | | 1.28 | | -2.19 | |  |
|  | 0.5 | 0.493 ±0.007 | | | 1.36 | | -1.50 | |  |
|  | 1.5 | 1.547 ± 0.018 | | | 1.17 | | 3.13 | |  |
| **MP** | 50 | 49.423 ± 0.593 | | | 1.20 | | -1.15 | |  |
|  | 100 | 96.220 ± 1.232 | | | 1.28 | | -3.78 | |  |
|  | 0.5 | 0.490 ± 0.005 | | | 1.04 | | -1.97 | |  |
|  | 1.5 | 1.516 ± 0.011 | | | 0.73 | | 1.08 | |  |
| **IMID** | 50 | 49.989 ± 0.459 | | | 0.92 | | -0.02 | |  |
|  | 100 | 93.472 ± 0.826 | | | 0.88 | | -6.53 | |  |
|  | 0.5 | 0.497 ± 0.005 | | | 1.01 | | -0.52 | |  |
|  | 1.5 | 1.497 ± 0.006 | | | 0.42 | | -0.20 | |  |
| **BPA** | 50 | 49.586 ± 0.602 | | | 1.22 | | -0.83 | |  |
|  | 100 | 98.594 ± 1.377 | | | 1.40 | | -1.41 | |  |
|  | 0.5 | 0.492 ± 0.008 | | | 1.57 | | -1.66 | |  |
|  | 1.5 | 1.499 ± 0.020 | | | 1.32 | | -0.10 | |  |
| **IBU** | 50 | 49.578 ± 0.839 | | | 1.69 | | -0.84 | |  |
|  | 100 | 99.333 ± 1.785 | | | 1.80 | | -0.67 | |  |
|  | 0.5 | 0.486 ± 0.010 | | | 1.96 | | -2.83 | |  |
|  | 1.5 | 1.499 ± 0.021 | | | 1.47 | | -0.09 | |  |
| **TCS** | 50 | 49.568 ± 0.621 | | | 1.25 | | -0.86 | |  |
|  | 100 | 97.273 ± 1.300 | | | 1.34 | | -2.73 | |  |

**Table S4:** Estimated values to evaluate the accuracy and precision of the method, inter-day, during 3 consecutive days.

|  | Theoretical concentration  (µg/mL) | Inter-day (n=18) | |  | |  |
| --- | --- | --- | --- | --- | --- | --- |
| Analytes |  | Measured concentration mean ± SD (µg mL^-1^) | Precision %RSD | | Accuracy %BIAS | |
|  | 0.5 | 0.490 ± 0.02 | 0.48 | | -1.91 | |
|  | 1.5 | 1.483 ± 0.024 | 1.59 | | -1.14 | |
| **PAR** | 50 | 49.283 ± 0.143 | 0.29 | | -1.43 | |
|  | 100 | 97.906 ± 0.198 | 0.20 | | -2.09 | |
|  | 0.5 | 0.492 ± 0.001 | 0.25 | | -1.62 | |
|  | 1.5 | 1.511 ± 0.03 | 2.21 | | 0.70 | |
| **MP** | 50 | 49.401 ± 0.129 | 0.26 | | -1.20 | |
|  | 100 | 96.253 ± 0.143 | 0.15 | | -3.75 | |
|  | 0.5 | 0.490 ± 0.001 | 0.14 | | -2.00 | |
|  | 1.5 | 1.496 ± 0.018 | 1.19 | | -0.28 | |
| **IMID** | 50 | 50.056 ± 0.188 | 0.38 | | 0.11 | |
|  | 100 | 93.694 ± 0.201 | 0.21 | | -6.31 | |
|  | 0.5 | 0.4968 ± 0.004 | 0.76 | | -0.64 | |
|  | 1.5 | 1.477 ± 0.018 | 1.21 | | -1.53 | |
| **BPA** | 50 | 49.505 ± 0.123 | 0.25 | | -0.99 | |
|  | 100 | 98.684 ± 0.102 | 0.10 | | -1.32 | |
|  | 0.5 | 0.496 ± 0.004 | 0.79 | | -0.75 | |
|  | 1.5 | 1.488 ± 0.013 | 0.86 | | -0.79 | |
| **IBU** | 50 | 50.175 ±0.518 | 1.03 | | 0.35 | |
|  | 100 | 100.798 ± 1.280 | 1.27 | | 0.80 | |
|  | 0.5 | 0.483 ± 0.005 | 1.07 | | -3.44 | |
|  | 1.5 | 1.464 ± 0.031 | 2.14 | | -2.42 | |
| **TCS** | 50 | 48.997 ± 0.587 | 1.20 | | -2.01 | |
|  | 100 | 96.335 ± 0.903 | 0.94 | | -3.66 | |


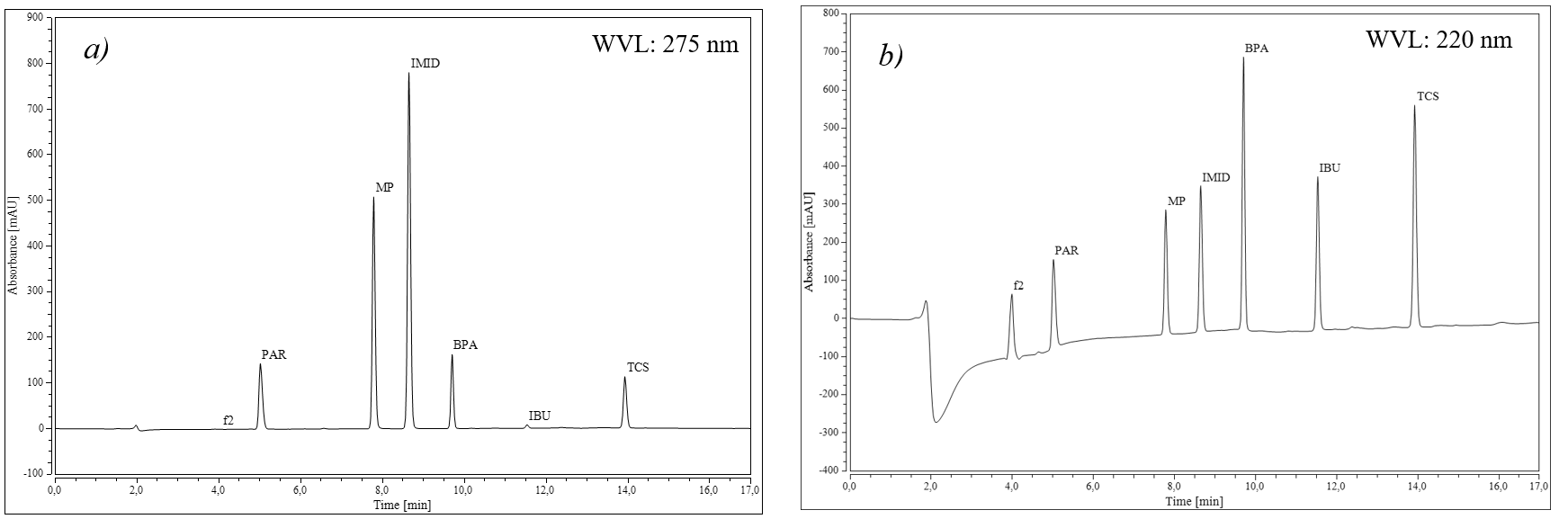


**Figure S1:** Chromatograms obtained to evaluate the selectivity and specificity of the method. a) and b) represent the six analytes diluted in the f2 medium, showing no matrix effect, at 275nm and 220 nm, respectively.

**Table S5:** Percentage of recovery of PAR, MP, IMID, BPA and IBU from the f2 culture medium (n=6).

|  | **PAR** | | | **MP** |  | | **IMID** | | | **BPA** | | | **IBU** | | | **TCS** | | |
| --- | --- | --- | --- | --- | --- | --- | --- | --- | --- | --- | --- | --- | --- | --- | --- | --- | --- | --- |
| μg mL^−1^ | **1** | **50** | **100** | **1** | **50** | **100** | **1** | **50** | **100** | **1** | **50** | **100** | **1** | **50** | **100** | **1** | **10** | **20** |
| %recovery | 92.1 | 95.2 | 94.6 | 92.4 | 96.1 | 93.7 | 92.4 | 101.8 | 92.7 | 84.6 | 90.2 | 92.4 | 95.4 | 92.6 | 85.1 | 49.2 | 20.7 | 3.85 |
